# Antibacterial Packaging for Cheese Based on Carboxymethyl Cellulose Composite with Zinc Oxide and Thyme Essential Oil

**DOI:** 10.3390/foods15101724

**Published:** 2026-05-14

**Authors:** Ludmila Motelica, Ovidiu-Cristian Oprea, Anton Ficai, Roxana Doina Trusca, Denisa Ficai, Catalina-Elena Constantin, Alina Maria Holban, Gabriel Mustatea, Elena Loredana Cirstoiu (Ungureanu), Carmen Curutiu

**Affiliations:** 1 Research Institute of the University of Bucharest (ICUB), University of Bucharest, Spl. Independentei 91-95, 0500957 Bucharest, Romania; ludmila.motelica@upb.ro; 2 Research Center for Advanced Materials, Products and Processes, National University of Science and Technology POLITEHNICA Bucharest, Splaiul Independentei 313, 060042 Bucharest, Romania; 3 National Research Center for Micro and Nanomaterials, Faculty of Chemical Engineering and Biotechnologies, National University of Science and Technology POLITEHNICA Bucharest, Splaiul Independentei 313, 060042 Bucharest, Romania; anton.ficai@upb.ro (A.F.);; 4 Academy of Romanian Scientists, Ilfov Street 3, 050044 Bucharest, Romania; 5 Faculty of Chemical Engineering and Biotechnologies, National University of Science and Technology POLITEHNICA Bucharest, Gh. Polizu 1-7, 011061 Bucharest, Romania; 6 Faculty of Biology, University of Bucharest, 030018 Bucharest, Romaniaalina.m.holban@bio.unibuc.ro (A.M.H.); carmen.curutiu@bio.unibuc.ro (C.C.); 7 National R&D Institute for Food Bioresources—IBA Bucharest, Dinu Vintila Street 6, 021102 Bucharest, Romania; gabi.mustatea@bioresurse.ro (G.M.); elena.ungureanu@bioresurse.ro (E.L.C.)

**Keywords:** carboxymethyl cellulose, fluorescence, ZnO, biodegradable, packaging, thyme, antibacterial, cheese, synergy

## Abstract

The food-packaging sector is undergoing a major transition driven by the environmental burden associated with petroleum-based plastics and the increasing demand for sustainable alternatives. In this context, biodegradable packaging materials capable of extending food shelf life through active preservation functions have attracted considerable interest. Cellulose is the most abundant natural polymer and an attractive candidate for sustainable packaging; however, it lacks intrinsic antimicrobial activity. In the present study, innovative carboxymethyl cellulose (CMC)-based composite films were developed by incorporating zinc oxide (ZnO) nanoparticles (NPs) and thyme essential oil (TEO) as antibacterial active agents. The obtained films exhibited strong antibacterial activity against both *Escherichia coli* and *Staphylococcus aureus*, completely eliminating planktonic cell viability after 3 h of contact and producing inhibition zones of up to 30 mm. In addition to their biological performance, the composite films showed improved mechanical and functional properties. ZnO NPs appear to act as multifunctional junctions within the CMC matrix, while the dispersed TEO droplets contribute, together with the inorganic phase, to reduced water-vapor transfer. The films retained good transparency in the visible range while exhibiting UV-A transmittance below 7%, indicating enhanced light-barrier performance. Preliminary tests on soft cheese indicated shelf-life extension up to 14 days at 4 °C, while in inoculated cheese slices packed in the composite films, *S. aureus* was not detected from the 3rd day. Overall, these results demonstrate the potential of CMC/ZnO/TEO composite films as biodegradable active packaging materials for perishable food products.

## 1. Introduction

Food packaging has become a major research priority because it lies at the intersection of food safety, shelf-life extension, and sustainability. Conventional petroleum-based plastics still dominate the packaging sector owing to their low cost and favorable mechanical and barrier properties; however, their persistence in the environment and their large contribution to post-consumer waste have intensified the search for safer and more sustainable alternatives. At the same time, conventional packaging does not provide intrinsic antimicrobial protection and therefore cannot directly limit microbial spoilage. For this reason, increasing attention has been directed toward packaging materials obtained from renewable sources that combine environmental compatibility with active preservation functions, particularly antimicrobial activity [1,2].

Polysaccharides are among the most attractive classes of biopolymers for sustainable packaging because they are widely available, renewable, and biodegradable [3,4]. Among them, cellulose is especially appealing because it is the most abundant natural polymer and can be obtained from wood, cotton, leaves, and other plant-derived sources, as well as from microbial production systems [5,6]. Structurally, cellulose is a linear polysaccharide composed of β-(1 → 4)-linked D-glucose units, and it has long been explored for packaging-related applications [7,8]. Through chemical engineering, various branched cellulose derivatives can also be obtained. Among its derivatives, carboxymethyl cellulose (CMC) is particularly useful because of its water solubility, good film-forming ability, and broad availability [9,10]. Nevertheless, cellulose-based matrices do not exhibit inherent antimicrobial activity; therefore, the development of antibacterial cellulose-based composites requires the incorporation of supplementary active agents such as inorganic nanoparticles, plant extracts, essential oils, antimicrobial peptides, or other bioactive compounds [11,12,13].

Zinc oxide (ZnO) is one of the most extensively studied inorganic antimicrobial agents, alongside silver-based nanomaterials. ZnO nanoparticles (NPs) exhibit strong antibacterial activity, which is influenced by particle size, morphology, and other physicochemical characteristics [14]. Their antimicrobial action is generally associated with a combination of reactive oxygen species (ROS) generation, direct damage to the bacterial cell envelope during nanoparticle–cell interactions, and Zn^2+^ release, all of which can impair membrane integrity and cellular metabolism [14]. In addition, ZnO is considered an attractive candidate for food-packaging applications because of its antimicrobial efficacy and its contribution to UV-screening performance. These characteristics explain why ZnO NPs are frequently selected as multifunctional additives in active food-packaging formulations [15,16].

Thyme is a culinary and medicinal plant with a long history of use in traditional medicine and food-related applications [17]. Thyme essential oil (TEO) is a natural antimicrobial extract owing to its rich composition in bioactive volatile compounds such as thymol, borneol, carvacrol, p-cymene, and γ-terpinene [18,19,20]. These constituents are associated with the well-documented antibacterial and antifungal properties of thyme and its derivatives [18,21]. In particular, thymol and carvacrol are lipophilic compounds that can disrupt the structure and permeability of microbial membranes, induce leakage of intracellular constituents, alter membrane potential, and interfere with essential metabolic processes, ultimately leading to growth inhibition or cell death [22]. Because of this broad biological activity and their relevance for food-related applications, thyme-derived compounds are of considerable interest in the design of natural active packaging systems [20,22].

In this study, we developed a biodegradable packaging material based on carboxymethyl cellulose (CMC), in which ZnO NPs and TEO were combined in order to provide antibacterial functionality together with improved barrier performance. To the best of our knowledge, the simultaneous incorporation of TEO and ZnO NPs into a CMC-based film and evaluation of its potential application as packaging have not been studied previously. Searching the Clarivate database by keywords “cellulose” AND “ZnO” yields just over 2000 entries, while using “cellulose” AND “thyme” returns 142 hits. However, no report could be identified for the keywords “cellulose” AND “ZnO” AND “thyme”, which represents a significant research gap in the area of antimicrobial biodegradable packaging. The rationale of this design is that the simultaneous incorporation of an inorganic nano-antimicrobial agent and a plant-derived essential oil may enhance antibacterial efficacy by exploiting complementary mechanisms of action [22,23]. At the same time, the individual components of the composite system are represented by a cellulose derivative used in food-related applications [24], a spice-derived natural extract, and ZnO as an inorganic active phase, making this approach relevant for the development of safer and lower-impact packaging materials. Accordingly, the objective of this work was to prepare and characterize CMC-based composite films containing ZnO NPs and TEO, and to evaluate their structural, morphological, thermal, optical, and barrier properties. Additionally, we have investigated their applicability in a real food system (soft cheese) preservation during storage at 4 °C, exploring the antibacterial properties of films to extend the quality characteristics of soft cheese, as measured by microbiological analysis. The obtained results will serve as a valuable reference for the development of new biodegradable antimicrobial packaging.

## 2. Materials and Methods

Zinc acetate, Zn(CH_3_COO)_2_·2H_2_O, glycerol (C_3_H_8_O_3_), thyme essential oil (TEO), n-butanol, carboxymethyl cellulose, Tween 80 and citric acid were purchased from Sigma Aldrich (Redox, Bucharest, Romania). ZnO NPs were prepared as previously reported in [25]. Briefly, 2.5 g Zn(CH_3_COO)_2_·2H_2_O were dissolved in 50 mL n-butanol and kept under reflux. After centrifugation and repeated washing with ethanol, ZnO NPs with a polyhedral shape and a size of ~20 nm were obtained.

### 2.1. Preparation of Nanocomposite Film

**Figure 1:**
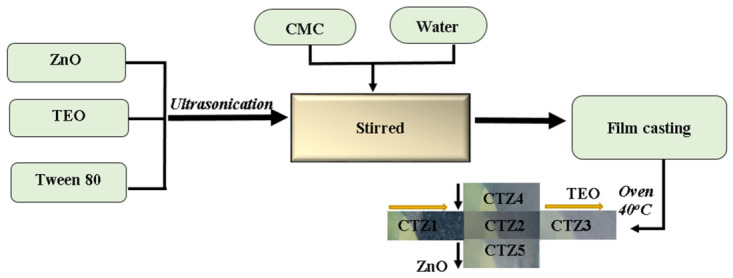
Schematic preparation flux for CTZ1-5 composite films.

The complete preparation procedure is depicted in Figure 1. Briefly, a quantity of 5 g carboxymethyl cellulose (CMC) was dissolved under stirring in 100 mL of water. A volume of 2 mL of glycerol was added as a plasticizer. Separately, a suspension of ZnO NPs with TEO was prepared in a 10 mL Tween 80 solution (concentration 10%) by ultrasonication.

The CMC, ZnO and TEO quantities together with the sample codifications are presented in Table 1. We added 0.1 g citric acid as a reticulation agent in each case. The solution was stirred for 30 min; then, it was degassed by ultrasonication for 15 min in order to eliminate all the bubbles. The composition was poured into Petri dishes and left to dry in the oven at 40 °C for 12 h. The dried films were peeled carefully and were further stored in plastic zip bags.

### 2.2. Characterization Techniques

#### 2.2.1. Fourier Transform Infrared Spectroscopy (FTIR) and Microscopy Measurements

A Nicolet iS50 FTIR spectrometer (Thermo Fisher Scientific Inc., Waltham, MA, USA), equipped with an ATR module and a DTGS detector, was used to record the spectra in the wavenumber range of 400 to 4000 cm^−1^. Each spectrum was obtained by averaging 32 scans at a resolution of 4 cm^−1^. FTIR maps were recorded with a Nicolet iS10MX FTIR microscope (Thermo Fisher Scientific Inc., Waltham, MA, USA), from 4000 to 650 cm^−1^.

#### 2.2.2. Ultraviolet-Visible (UV-Vis) Spectra Determination

Ultraviolet-Visible (UV-Vis) spectra were recorded in the range of 200–900 nm with a JASCO V560 spectrophotometer (Jasco Inc., Easton, PA, USA), in diffuse reflection mode. The spectrometer was equipped with a 60 mm integrating sphere and a film holder. Opacity values were calculated following the method described in [26], using Equation (1), where A_600_ represents the absorbance value at 600 nm, and x represents the average film thickness in millimeters (measured in three positions across a 20 mm × 20 mm sample):

(1) Opacity = A_600_/x,

A higher opacity value indicates that the film is less transparent.

#### 2.2.3. Photoluminescence (PL) Spectra Measurements

The photoluminescence (PL) spectrum was recorded using a Perkin Elmer LS55 spectrometer (Waltham, MA, USA), using an excitation wavelength of 320 nm, a cutoff filter of 350 nm, a scanning speed of 200 nm × min^−1^ and 10 nm slits.

#### 2.2.4. Scanning Electron Microscopy (SEM)

The surface morphology and microstructure of the films were investigated by scanning electron microscopy (SEM) using QUANTA INSPECT F50 equipment (FEI Company, Eindhoven, The Netherlands). The device is equipped with an energy-dispersive X-ray spectrometer (EDS) that was used to map the distribution of the elements in the samples.

#### 2.2.5. Simultaneous Thermal Analysis (STA) Measurements: Thermogravimetry (TG) Coupled with Differential Scanning Calorimetry (DSC)

Thermal stability was monitored with a STA 449C Jupiter, TG-DSC system from Netzsch (NETZSCH-Gerätebau GmbH, Selb, Germany), between 20 and 900 °C, under an air flow of 50 mL/min.

#### 2.2.6. Water Vapor Permeability (WVP) Measurements

Water vapor permeability (WVP) was evaluated according to the method described in [27], using glass flacons (1.2 cm diameter) sealed with the test film. Each flacon contained 1.1 g anhydrous CaCl_2_. The sealed flacons were placed in a desiccator at 25 °C and 100% relative humidity (RH). At predetermined time intervals, each flacon was weighted to quantify the mass of water vapor transmitted through the films. WVP values were then calculated by using Equation (2):

(2) $$WPV=\;\frac{\left(M_{t}-M_{0}\right)}{∆t\times ∆P\times S}\times L,$$

where *M*_0_ and *M_t_* are the flacons’ masses at the initial moment and at time *t*, *L* is the film thickness (m), *S* is the film area at the flacons’ opening (m^2^), Δ*P* is the difference in water vapor pressure between the film sides at the temperature of 25 °C and a RH gradient of 100% (3169 Pa), and Δ*t* is the time (s). The measurements were performed in triplicate.

#### 2.2.7. Mechanical Properties of the Films

The mechanical properties, such as tensile strength (TS), elongation at break (EB) and Young’s modulus, were determined using a Universal Testing Machine (Instron Testing System Model 5965, Norwood, MA, USA), equipped with a 5 kN load cell, consistent with guidelines stated in the ISO 527-1:2019 [28] and ISO 527-3:2018 [29] methods, using a specimen type 1B. The initial grip separation (gauge length) was set to 50 mm and stretched with a crosshead speed of 50 mm/min. Three samples were measured for each film. The thickness of the samples was measured by using a calibrated micrometer (Vogel Germany) in five random positions around the sample. *TS* (MPa) and *EB* (%) were calculated using Equations (3) and (4):

(3) $$TS\;(MPa)=\;\frac{F_{max}}{A},$$

(4) $$EB\;(\%)=\frac{L_{2}-L_{1}}{L_{1}},$$

where *F_max_*, *A*, *L*_1_ and *L*_2_ are the maximum load (N), initial cross-sectional area (m^2^), initial and final length of the sample. The Young’s modulus (MPa) values were determined from the slope of the elastic region within the stress–strain curves.

#### 2.2.8. Packaging Tests on Cheese

Cuboidal cheese samples, with edges of ~20 mm, were packed in the composite CTZ films and placed at 4 °C and 75% RH (in a refrigerator) for two weeks. Samples were measured daily to establish the weight loss as a percentage of the initial mass. The pH values were determined initially and after two weeks of storage. All samples were tested in triplicate.

#### 2.2.9. Antimicrobial Qualitative Assessment

For antimicrobial activity, two reference bacterial strains were used: one Gram-positive strain—*Staphylococcus aureus* ATCC 25923 (*S. aureus*)—and one Gram-negative strain—*Escherichia coli* ATCC 25922 (*E. coli*). The assay was based on a modified Kirby–Bauer disk diffusion method. From 24 h culture on nutrient agar, bacterial colonies were suspended in sterile physiological saline (SPS) until 0.5 McFarland microbial suspensions (1.5–3 × 10^8^ CFU/mL) and UV sterilized spread over the surface of nutrient medium using a sterile swab. Subsequently, the samples were placed and the plates were incubated for 18–24 h at 37 °C. The bacterial growth inhibition zones surrounding each sample were measured and the results were described as the diameter of the inhibition zone, expressed in mm. All experiments were performed in triplicate.

#### 2.2.10. Quantitative Analysis of Antimicrobial Effect

The samples were utilized to evaluate their antibacterial effects against free-floating (planktonic) microbial cells. Briefly, an individual sample was placed in each well of a 24-well sterile plate. In these wells, 1 mL of liquid medium (nutritive broth) was added, followed by 10 μL of microbial suspension at a density of 0.5 McFarland, prepared in sterile physiological saline. The 24-well plates thus prepared were incubated at 37 °C for 24 h. After different periods of the incubation time (2 h, 4 h, 8 h and 24 h), serial decimal dilutions in sterile physiological water were made and 10 μL of each dilution was inoculated in duplicate on nutrient agar media to obtain and quantify the number of colony-forming units/mL (CFU/mL).

#### 2.2.11. Microbiological Analysis of In Vivo Packed Cheese Slices

Freshly cut cheese slices of ~5 mm thickness (of ~5 g) were exposed to UV light for 20 min on both sides to ensure sterility. Samples were inoculated by the addition of previously prepared *S. aureus* cell suspension, obtaining a 5 log CFU/g concentration in cheese. After that, the packing films were applied in contact with the inoculated samples. The slices were stored for 14 days at 4 ± 1 °C, being analyzed at 0, 3, 7 and 14 days. Samples were placed in stomacher bags containing 0.1% peptone water and stomached for 90 s. Decimal dilutions were prepared and inoculated on Baird-Parker (BP) agar plates and incubated at 37 °C for 48 h. The results were expressed as log CFU/mL for *S. aureus*. All experiments were performed in triplicate.

#### 2.2.12. Statistical Analysis

The obtained results were statistically analyzed using the analysis of variance (ANOVA) performed with Microsoft Excel 2016 (Microsoft Corp., Redmond, WA, USA), having installed XLSTAT 2020.5.1 add-on. The Shapiro–Wilk test was used to check the normal distribution of the data; by Levene’s test, we assessed the homoscedasticity of the residuals; and the results were compared by Tukey’s (HSD) test, so that the pairs of films that differed in terms of statistical significance were revealed (where *p* < 0.05).

## 3. Results and Discussion

### 3.1. FTIR Spectroscopy and Microscopy

**Figure 2:**
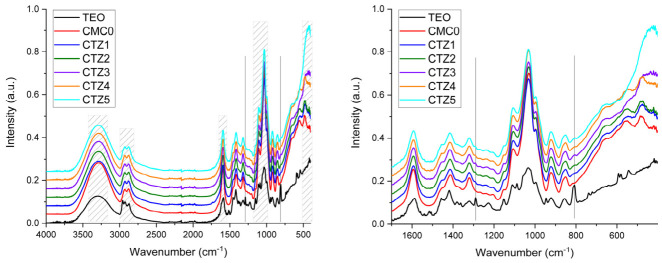
FTIR spectra for TEO, CMC0, and CTZ1-CTZ5 composite films and zoom-in.

By using Fourier transform infrared spectroscopy (FTIR) in the wavenumber range of 4000–400 cm^−1^, we investigated the presence of certain functional groups and the interactions between some components of the nanocomposite films (Figure 2).

The FTIR analysis of the TEO and films highlights several key characteristic absorption bands that define components of the chemical structure. A broad band at 3288 cm^−1^ is attributed to the stretching vibrations of hydroxyl groups, indicating intermolecular and intramolecular hydrogen bonding, both in CMC and in TEO components like thymol, carvacrol, and borneol [30,31]. The bands between 2870 and 2927 cm^−1^ correspond to the carbon–hydrogen bonds in the –CH_2_ groups of the cellulose backbone, namely, the asymmetric and the symmetric stretching vibrations. For TEO, an additional peak at 2964 cm^−1^ corresponding to the asymmetric C-H vibration in -CH_3_ can be noticed, as many compounds have this moiety. The peak at 1592 cm^−1^ represents the asymmetric stretching vibrations of the carboxyl groups, a fingerprint in CMC along the peak from 1416 cm^−1^ assigned to the symmetric stretching vibration of the –COO^-^ group [32,33,34]. Very close to it, at 1596 cm^−1^, the aromatic –C=C-stretching vibration can be observed in TEO’s FTIR spectrum, while the peak from 1418 cm^−1^ can be assigned to the in-plane deformation (bending vibration) from the isopropyl moiety bonded to the aromatic ring in thymol [35,36]. The band from 1319 cm^−1^ is assigned to –OH bending vibration and/or –CH_2_ wagging [37,38]. In the FTIR spectrum of TEO, a small peak at 1289 cm^−1^ is not overlapping with any CMC bands, being assigned to the aromatic C-O-H stretching vibration (from phenolic compounds) [39]. This peak, although with smaller intensity, can be noticed in all composite films, CTZ1-CTZ5, as a direct indication of TEO presence. In the composite films, the highest intensity for this peak can be observed for the CTZ3 sample that contains the largest TEO quantity. A second characteristic peak that can be identified only in TEO’s FTIR spectrum lies at 807 cm^−1^ and can be attributed to out-of-plane C-H wagging vibrations of the aromatic rings from thymol, p-cymene and carvacrol [40,41]. The very strong absorption band at 1030 cm^−1^ (often in the 1000–1100 cm^−1^ range) is associated with the stretching vibrations of the carbon–oxygen (C-O) bonds in the polysaccharide ring and ether linkages (C-O-C pyranose ring skeletal vibration) [42,43,44]. In the case of TEO, the 1034 cm^−1^ can also be assigned to the C-O stretching vibration in the phenolic groups [45].

The strong absorption band in the domain 400–500 cm^−1^ is assigned to the Zn-O stretching vibration [46].

**Figure 3:**
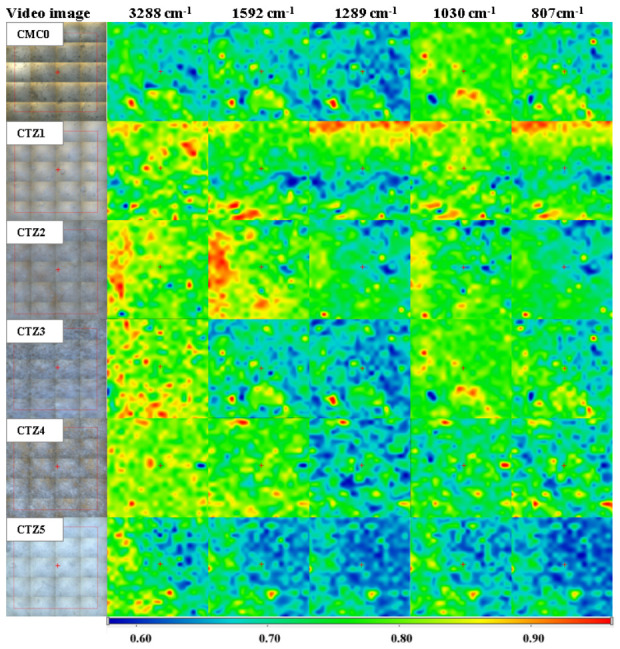
The FTIR maps for carboxymethyl cellulose (CMC0) and CTZ1–CTZ5 films at wavenumbers 3231 cm^−1^, 1591 cm^−1^, 1289 cm^−1^, 1034 cm^−1^ and 809 cm^−1^.

The FTIR microscopy maps are presented in Figure 3. The maps were recorded at specific wavenumbers, characteristic of CMC and TEO, as mentioned above. The 3288 cm^−1^ corresponding to the –OH stretching vibration, 1592 cm^−1^ from the -COO^-^ groups or aromatic C=C and the 1030 cm^−1^ from the C-O bonds are common for CMC and TEO, while 1289 cm^−1^ assigned to the aromatic C-O-H stretching vibration and 807 cm^−1^ from the out-of-plane C-H wagging vibrations of the aromatic rings are specific to the TEO compounds [47]. The FTIR maps indicate that the samples are quite uniform, with good homogeneity; the ZnO and TEO are well dispersed into the polymeric matrix. The FTIR maps have a similar appearance for each sample, with only minor differences between maps acquired for common and specific wavenumbers, with no major distribution gradient for TEO.

### 3.2. UV-Vis and PL Spectrometry

**Figure 4:**
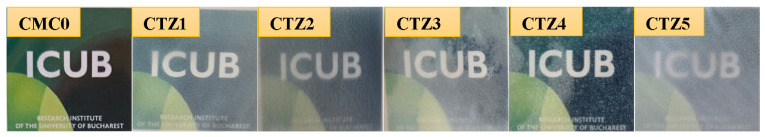
The visual appearance and transparency of CMC0 and CTZ1-CTZ5 films.

The obtained films are quite transparent, as indicated by the visual appearance and the transparency of the CMC0 and CTZ1-CTZ5 composite films, as depicted in Figure 4. The sample with the lowest transparency is CTZ5, which contains the largest quantity of ZnO NPs, which act as a physical barrier for the light. Consequently, the highest transparency is exhibited by the CTZ4 sample. The transparency of a packaging film is an important property, as customers are very interested in being able to see the packed foodstuffs in general.

The quantification of the transparency/opacity of the films is done as indicated in [26], by Equation (1), with the results presented in Table 2.

The composite films with the largest amount of TEO or ZnO, CTZ3 and CTZ5, are statistically different from the other films, which do not differ among them from an opacity point of view. The opacity values indicate that the films have a high transparency, comparable with other cellulose-based composites. For bacterial cellulose films with epigallocatechin-3-gallate, a recent paper reports values in the range of 0.113–0.231 [48], lower than the 0.28–0.85 values reported for alginate-based composites in [26,49].

**Figure 5:**
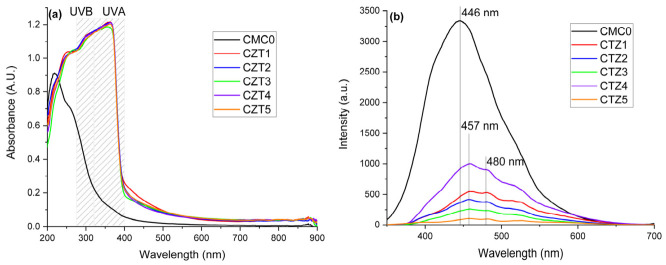
The UV-Vis (**a**) and PL (**b**) spectra for CMC0 and CTZ1-CTZ5 composite films, with the UVA and UVB domains.

The spectra for the CMC0 and composite films are presented in Figure 5a, and are very close to the ideal behavior for the packaging films, with good visible transparency and UV-blocking properties. It can be seen that while the visible absorbance marginally increased, the films became highly opaque to both UVA (320–400 nm) and UVB (280–320 nm). The absorbance values in the visible region indicate a very good transparency of the films when compared with CMC-based composites with ZnO and plant extracts previously reported [50]. The slight increase in absorbance in the violet-blue region of visible spectra is responsible for the yellowish tint of the composite films. The UV blocking capacity is mainly generated by the ZnO NPs’ presence, and is very effective even at lower concentrations, due to its intrinsic properties. ZnO is well known for its capacity to absorb and scatter UV radiation, and its applications in cosmetics as sun-screen [51]. The blocking of the UV radiation by the packaging film is very important, as these high-energy photons are responsible for the initiation of the photo-oxidation reaction of lipids, vitamins and other essential nutrients from the foodstuff.

The fluorescence of the films is presented in Figure 5b. The simple CMC0 film exhibits a very high fluorescent emission, which is characteristic of cellulose [27,52,53] and other polysaccharides like chitosan [54,55,56], alginate [26,57] or starch [58]. Traditional luminogens usually exhibit a bright emission in dilute solutions but emit dimly or even with no light at all when aggregated or in the solid state, which is known as the aggregation-caused quenching [59]. A new fluorescence mechanism was first reported in 2001 [60] in a different substance class. In this case, a negligible emission in dilute solutions was observed, but enhanced fluorescence occurred in the aggregated or solid state, which was termed aggregation-induced emission (AIE), with polysaccharides being one class of the AIE luminogens [61]. A common feature of these AIE molecules is that they are non-conjugated and have many electron-rich heteroatoms, like oxygen or nitrogen [62,63]. We previously reported the strong fluorescence for pure cellulose manual paper samples from the XVII-XIX centuries [52,53], but also for cellulose derivatives [27], in line with previous findings like those in [64], where authors stated that the origin of cellulose fluorescence remains uncertain, but underlines the importance of the solid state for fluorescence study. There are studies that report even phosphorescence beside fluorescence of cellulose, starch, etc. [65], but always in the solid state, indicating the fundamental contribution of the close packaging interactions among polymer chains. Intermolecular interactions generated by the close proximity of the polymer chains are effective in decreasing the vibrational dissipations of the excitons and, therefore, helpful for the light emission. Electron-rich oxygen and/or glucose units are accountable for the unique emission behaviors. Although there is no classic chromophore, there are numerous electron-rich oxygen-containing groups with lone pair electrons. In the solid form, through proper contacts, such groups may form a variety of clusters, in which electron clouds of the oxygen groups are overlapped and shared, thus generating novel clustered electron-rich chromophores with lowered energy gaps and extended effective conjugation lengths. Meanwhile, effective molecular interactions significantly prevent non-radiative deactivation channels, thereby endowing such “clustered chromophores” highly emissive in the solid powder states [65].

The introduction of ZnO NPs, also known for their fluorescent violet-blue emission [66], and TEO leads to a dramatic decrease in the composite films’ fluorescence. The samples CTZ1-CTZ3 exhibit a moderate decrease in the emission intensity as the TEO quantity increases. At the same time, in the series CTZ4, CTZ2, and CTZ5, the fluorescence decreases more sharply as the ZnO quantity increases. This mechanism can be explained by the dispersion of the ZnO NPs among cellulose polymeric chains, breaking the previous aggregation state that was responsible for AIE. The TEO components also become dispersed into the CMC matrix and disturb the aggregation of the polymeric chains, but at the molecular level; therefore, their influence on AIE is smaller. Such a decrease in fluorescence, induced by the introduction of small molecules in polysaccharides matrix, was previously reported in [57]. At the same time, the presence of other molecules on the ZnO NPs’ surface (e.g., cellulose or TEO components) blocks the native ZnO fluorescence by offering non-radiative pathways for de-excitation.

### 3.3. Scanning Electron Microscopy (SEM) Results

**Figure 6:**
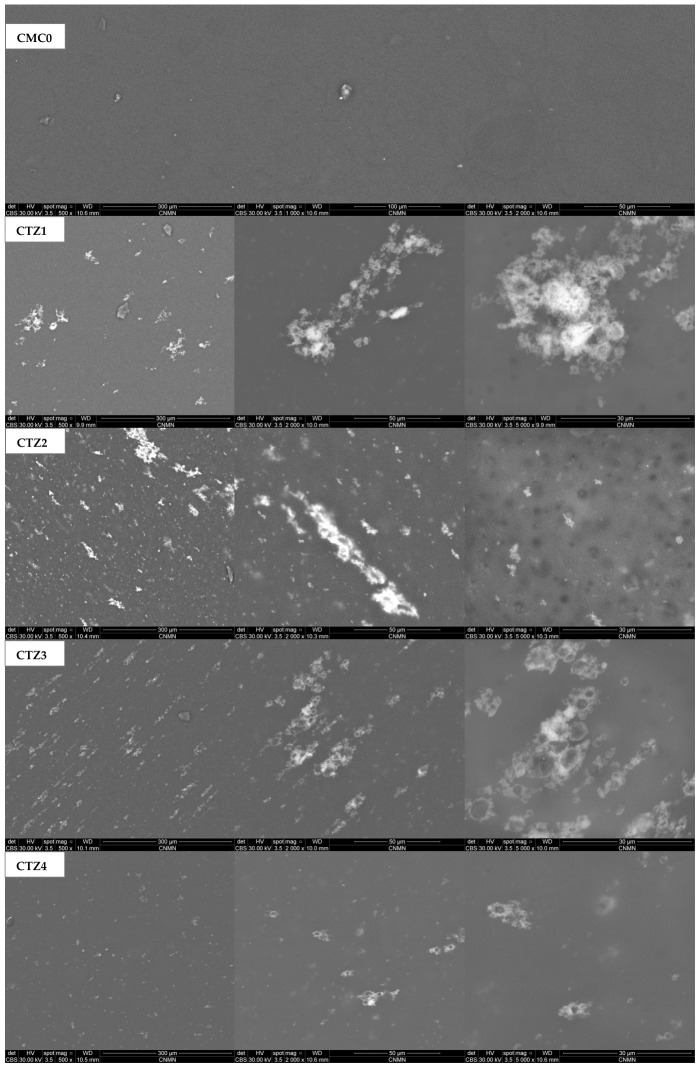

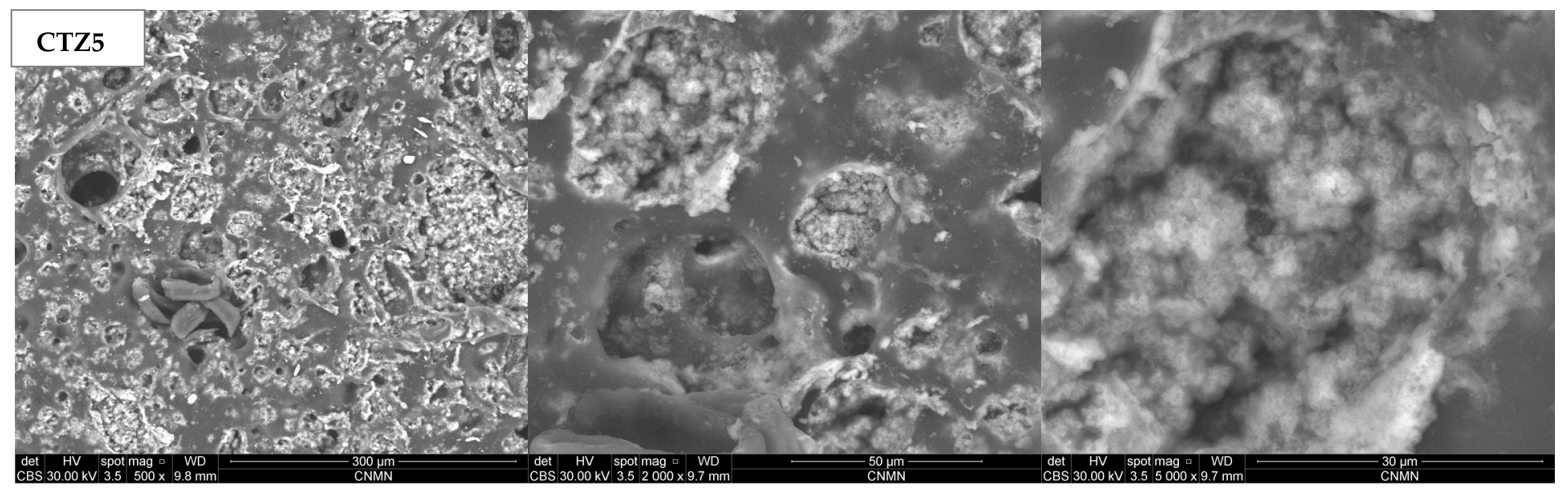
The SEM micrographs for CMC0 and CTZ1-CTZ5 composite films.

The morphology of the composite films was investigated further by scanning electron microscopy (SEM), with the results presented in Figure 6.

The influence of TEO and ZnO NPs incorporation in different ratios was also evaluated at different magnifications. As can be seen, the simple CMC0 has a neat surface, with compact morphology, characteristic of polymeric films obtained by the solvent casting method [67].

**Figure 7:**
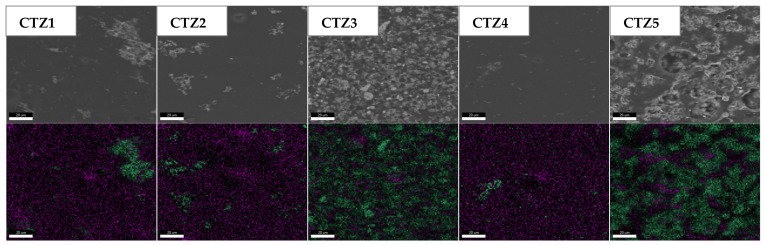
The EDX elemental maps for CTZ1-CTZ5 composite films (Zn-green, C-purple).

For the composite films, both TEO and ZnO NPs exert influence on the morphology. The increase in TEO quantity in the CTZ1-CTZ3 series leads to an increase in droplets’ dispersion into the CMC matrix at first, and then to an increase in the droplets’ size. Due to the surface tension, the edge of the droplets is covered with multiple ZnO NPs, generating local agglomerations [68]. The dispersion of ZnO NPs was assessed by energy-dispersive X-ray spectroscopy (EDX), with the results presented in Figure 7.

In the series CTZ4, CTZ2, and CTZ5, the sample with the smallest quantity of ZnO NPs presents the most uniform surface, with some random nanoparticle agglomeration being observed around larger TEO droplets. The least uniform surface is exhibited by the CTZ5 sample, where the presence of large surface pits and pores can be observed. These are covered with excess ZnO NPs, confirming the tendency of aggregation around TEO droplets. Nevertheless, the individual pores do not appear to be connected in a larger network or to penetrate the film thickness, and most likely represent surface defects generated by the TEO droplets. Such surface defects might affect both mechanical resistance and barrier properties. Surface pores decrease the local thickness of the film and, therefore, the water vapor pathway is shorter, with the values for WVP increasing. At the same time, a thinner film will have a lower mechanical resistance than a thicker one. Nevertheless, the values obtained for both WVP and mechanical properties indicate that such defects, although having a noticeable contribution, do not significantly impact the composites. The barrier and mechanical properties are still being improved for all the composite samples when compared to the neat CMC film. Similar porous structures were reported previously in the literature when plant extracts were used in combination with cellulose derivatives [67,69]. In the case of the CTZ5 sample, the ZnO NPs are found to be in the most continuous distribution at the surface of the film, which can lead to a stronger antimicrobial activity.

### 3.4. Thermal Analysis

**Figure 8:**
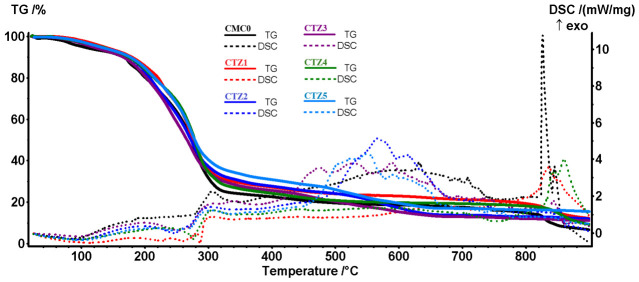
TG-DSC curves for CMC0 and CTZ1-CTZ5 composite films.

The thermal analysis results are presented in Figure 8. The thermogravimetric (TG) solid lines indicate the mass variation for each sample, while the associated thermal effects are visible on the differential scanning calorimetry (DSC) dotted curves. All samples have, in general, a similar thermal behavior. Up to 150 °C, the samples are losing residual water molecules trapped in the polymeric network and most volatile components from TEO. Between 150 and 340 °C, the CMC matrix is decomposed by losing structural water molecules, while some minor oxidation processes can occur, as indicated by the weak exothermic effects. Also, in this temperature interval, the high boiling point constituents of TEO (like thymol, borneol or carvacrol) are eliminated. After 340 °C, the carbonaceous residual mass generated by the CMC is burned away, with the process being accompanied by exothermic effects on DSC curves.

The principal numerical data presented in Table 3 permit a more detailed view of the composite samples’ thermal behavior. The T_1%_, T_5%_ and T_10%_ represent the temperature at which a sample lost 1%, 5% or 10% of its initial mass. As can be seen, the control sample CMC0 presents the lowest values for these temperatures, indicating a quick removal of the residual water. In the absence of ZnO NPs that act as a thermal stabilizer, the CMC is decomposed more easily, with the mass loss up to 340 °C having the highest value. Also, due to the absence of ZnO NPs, the residual mass has the lowest value. Similar behavior was reported previously for cellulose-based composites with inorganic fillers [43].

The samples CTZ1, CTZ2 and CTZ3 have an increasing amount of TEO, which will be removed during thermal analysis; therefore, the residual mass drops from 12.10 to 10.99%, but will be higher than that of the CMC0 sample due to the ZnO NPs’ presence. At low temperatures, we can see that increasing TEO content leads to the lowering of the T_1%_–T_10%_ values, as more volatile components can leave the samples.

In the samples CTZ4, CTZ2, and CTZ5, the ZnO NPs’ amount increases, which leads to an increase in the residual mass, from 9.23% to 15.30%. The presence of more ZnO NPs offers additional anchoring points for TEO components, leading to a minor increase in T_1%_–T_10%_ values. At the same time, the recorded mass loss at 340 °C decreases from 73.06 to 65.52% as the ZnO content increases. Overall, the presence of the inorganic filler, ZnO NPs, in the polymeric matrix leads to an increase in thermal stability, with similar findings being previously reported in the literature [70,71,72].

### 3.5. Water Vapor Permeability (WVP) Results

Another important feature for the packaging films is the water vapor permeability that is responsible for the loss of humidity from packed foodstuffs. In the case of dry food, a high WVP value will allow too many water molecules to permeate through the package and spoil the food. Therefore, for food applications, the composite films with the ability to block the water molecules will have a better preserving ability, maintaining a stable environment around the food and prolonging the shelf life [73].

Control film CMC0 exhibited the largest value for WVP, as expected for a hydrophilic polysaccharide that can form multiple hydrogen bonds with the passing water molecules, guiding them [74,75]. All the composite films exhibit an improvement in the water vapor barrier properties, with the WVP values decreasing due to the presence of TEO droplets and ZnO NPs (Table 2). Values of the same order of magnitude are reported in [76], where a simple CMC film exhibited a larger WVP value (2.37 × 10^−10^ g/Pa∙m∙s), which dropped after TEO incorporation to 0.12 × 10^−10^ g/Pa∙m∙s. On the other hand, CMC-based films have presented higher WVP values, which increased from 1.93 for a neat CMC film to 7.04 × 10^−10^ g/Pa∙m∙s for the composite with pomelo peel powder and nisin [67].

Essential oil droplets are highly hydrophobic and therefore block the water pathway. The ZnO NPs are impermeable and also act as impassable points in the water path. Moreover, the ZnO NPs are acting as cross-linking points for the polysaccharide chains, decreasing the space between them and blocking the polymer backbone in a more rigid network [51,74]. Additionally, some zinc ions can be released inside the polymer matrix and will create an even tighter “egg-box” structure [26,77].

**Figure 9:**
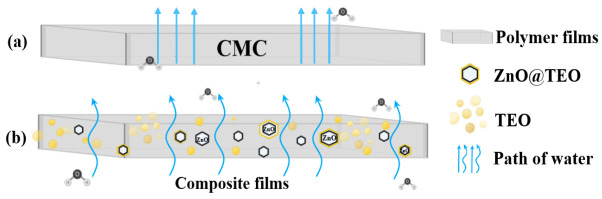
The proposed mechanism for water vapor permeability. Water molecule pathway through the plain CMC film (**a**) and a more tortuous pathway for the composite CTZ1-CTZ5 films (**b**).

Both TEO and ZnO NPs’ presence impose a longer and more tortuous pathway on the water molecules (Figure 9); therefore, the WVP values decrease [78]. The value obtained for the CTZ5 film indicates an aggregation effect from the ZnO, confirmed by SEM micrographs, that can generate surface defects and micro cracks into the composite structure [79].

### 3.6. Mechanical Characterization of the Films

The TS values indicate the maximum resistance of a sample to snapping while being under tension. Young’s modulus is an intrinsic value for the stiffness of a composite and is correlated to TS. Finally, the EB indicates the flexibility of a sample [77]. The mechanical properties of the CMC0 and composite films CTZ1-CTZ5 are presented in Table 2.

It can be observed that the mechanical properties of the composite films are enhanced when compared with the control CMC0 sample. The presence of ZnO NPs that act as additional cross-linking points leads to an increase in the TS and Young’s modulus vs. the neat polymer film, as reported for simple composites in [78,80]. On the other hand, the presence of TEO components leads to a slight decrease in the TS and Young’s modulus values in the CTZ1-CTZ3 series. This behavior is related to the disruption of the CMC matrix, with some of the hydrogen bonds among the polymer chains being reformed with molecules like thymol and carvacrol. At the same time, other hydrophobic components like p-cymene and γ-terpinene are entrapped among chains, disrupting the hydrogen bonds. A similar effect was previously reported in the literature for polysaccharide-based films with various natural extracts [81,82,83]. The same mechanism is responsible for the higher EB values, as the polymer chains become more mobile due to the TEO addition [84].

The modification of ZnO NPs’ amount while keeping TEO constant has mixed results on mechanical properties. In the case of the CTZ4 sample, the EB value is sharply decreasing, indicating that the ZnO NPs’ amount is not sufficient to counterbalance the effect of TEO, with a similar effect reported in [51,85]. At the same time, increasing the ZnO amount in sample CTZ5 leads to a high increase in Young’s modulus, indicating a stiffer composite, but with lower TS and EB values that can originate from ZnO NP-induced defects by local agglomerations, as indicated by SEM analysis.

### 3.7. Antibacterial Activity Assay

**Figure 10:**
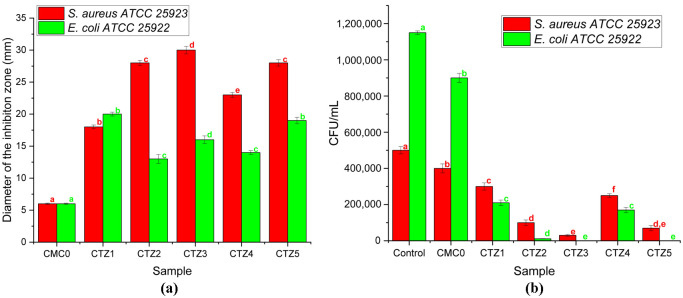
The diameter of the inhibition zone (**a**) and planktonic growth at 2 h (**b**) for CMC0 and CTZ1-CTZ5 composite films, against *S. aureus* ATCC 25923 (red) and *E. coli* ATCC 25922 (green). Values are means ± standard deviation for *n* = 3 measurements. Different letters of the same color indicate statistically significant differences between samples (*p* < 0.05).

The main purpose of the present study is to obtain antibacterial cellulose-based packaging films; therefore, the capacity to combat pathogen contamination was determined against *S. aureus* and *E. coli*. In Figure 10, the results from the determination of the diameter of the inhibition zone and colony-forming units/mL at two hours are presented.

Qualitative analysis showed that all samples have exhibited strong antibacterial activity for both analyzed species, with the diameter of the inhibition zone up to 30 mm for the CTZ3 composite film that has the highest TEO content (Figure 10a). Also, the results revealed that increasing the TEO content from CTZ1 to CTZ2 leads to a significant increase in the diameter of the inhibition zone from 18 to 28 mm, but further increase in TEO content in CTZ3 leads to a modest increase in the diameter to 30 mm, suggesting a capping of the antibacterial effect.

Quantitative analysis confirms the results. The viability of bacterial cells in contact with the composite films is presented in Figure 10b. The impairment of viability was clearly visible after 2 h, with the sample CTZ3 (that has the highest TEO concentration), but also CTZ2 and CTZ5 (that has high content of ZnO), exhibiting the most potent action for both strains, even a complete inhibition of growth in the case of *E. coli*. After 4 h of contact, no viable microbial cells were recovered, suggesting complete elimination of both *S. aureus* and *E. coli*. The significant decrease in bacterial viability can be related to the release and presence of the bioactive compounds in the growth media.

**Figure 11:**
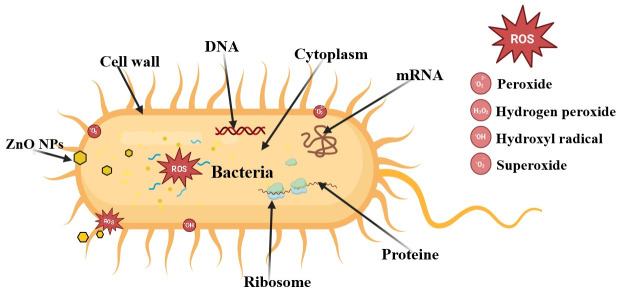
Proposed antibacterial mechanism for the ZnO NPs.

ZnO is effective against foodborne pathogenic bacteria such as *E. coli* and *S. aureus*, with its mechanism involving the generation of reactive oxygen species (ROS) that damage cellular structures (Figure 11). However, for ZnO, there are multiple mechanisms proposed in the literature, and it has been reported that the antimicrobial activity is also size and morphology-dependent [86]. While smaller nanoparticles tend to cluster near the cell’s membrane, they also have a larger specific surface and thus can produce more ROS.

The morphology plays a specific role when it comes to mechanical damage like puncture, abrasion or rupture of the membrane, leading to leakage of proteins, RNA or potassium ions and cell death [87]. The zinc ion released near the cell’s membrane or inside, after NP internalization, can contribute to microbial cell death by cytotoxicity, by binding essential components and nutrients of the cell [87]. Recently, it was also reported that ZnO NPs can downregulate the expression of Ag43 surface protein, responsible for biofilm formation and bacterial adherence, indicating a novel antimicrobial pathway for ZnO NPs [88].

TEO’s primary active components, like thymol and carvacrol, disrupt microbial cell walls, leading to cell death. There are multiple studies indicating that thymol reduces the virulence factor expression by interfering with quorum-sensing pathways, thus inhibiting biofilm formation [89,90]. The hydrophilicity of carvacrol and thymol is increased by the phenolic hydroxyl group, leading to microbial cell membrane dissolution and damage [91]. In a recent study, thymol exhibited stable target-specific interactions with essential microbial proteins, particularly FtsZ and sterol 14-α-demethylase, leading to strong antibiofilm activity [92].

One possible mechanism for the synergy is that thymol and carvacrol are sensitizing the cell membrane, making it more prone to the multiple antimicrobial mechanisms of ZnO NPs. The synergy between antimicrobial activities of ZnO NPs and TEO was reported before in [93], with the antimicrobial activity of ZnO NPs + TEO being the highest of the 10 combinations tested. The viability of the bacterial cells is dependent on the concentration of both TEO and ZnO NPs. Here, it can be clearly seen that increasing the TEO content in the series CTZ1-CTZ3 leads to stronger antibacterial activity against both strains, while increasing the ZnO NPs content in the series CTZ4, CTZ2, and CTZ5 also decreases the number of bacterial cells comparative with the control. The complete removal of *E. coli* by both CTZ3 and CTZ5 indicates that strong antimicrobial activity can be reached by increasing either of the two components, TEO or ZnO NPs, allowing a flexible composition that also takes into account the optimization of other parameters.

### 3.8. Evaluation of Packaging Films on Soft Cheese

**Figure 12:**
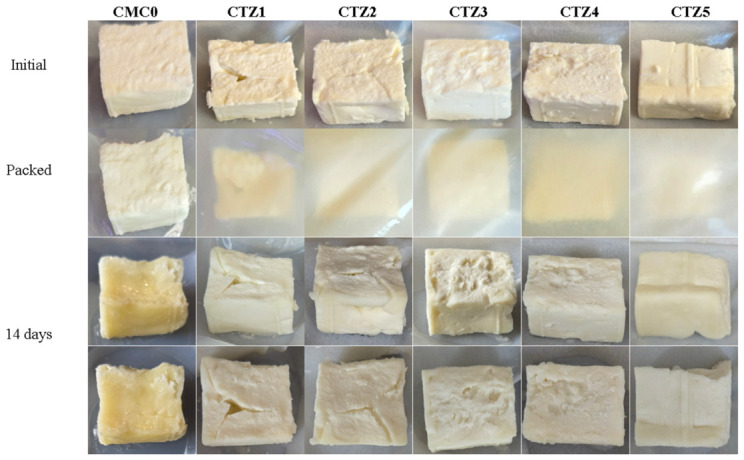
Visual appearance of soft cheese bits wrapped in CMC0 control film and CTZ1-CTZ5 composite films, initially and after 14 days of storage at 4 °C and 75% relative humidity.

Cheese is an important food derived from milk and consists mainly of proteins (casein), fats, mineral salts, water and some lactose traces. Soft cheese has a higher water content and, if unsalted, the rapid development of microorganisms leads to a short shelf life. Cuboidal samples of soft cheese, ~8–9 g, were weighted and wrapped in simple CMC or composite films, and then stored at 4 °C and 75% RH for 14 days. Finally, the samples were weighted, inspected visually and the pH was determined. The visual appearance of the cheese samples is depicted in Figure 12. While samples packed in composite films CTZ1-CTZ5 maintained the soft texture and white appearance, the control sample became harder and changed color to dark yellow. Similar modifications are reported in the literature when the cheese is spoiled [94].

The recorded mass loss values are presented as % of the initial mass in Table 4. Soft cheese has a high water content, and water vapors are eliminated into the environment continuously unless the packaging film blocks them. It can be observed that all samples packed in the composite films exhibit a lower rate of water loss when compared with the control sample. Moreover, the samples CTZ2, CTZ4 and CTZ5 have statistically similar values, while statistical differences can be observed among samples CTZ1, CTZ2 and CTZ3, indicating that the variation in TEO amount in the composite films has a larger influence than the variation in ZnO quantity [95].

The initial pH value for the cheese was 4.51, and for the samples packed in composite films CTZ1-CTZ5, it was found to be unmodified after 14 days. At the same time, the pH of the sample packed in the CTMC0 sample increased to 5.13, indicating a possible spoilage by microorganisms’ colonization. The pH value for the samples wrapped in the composite films remained stable, similar to previous reports in which spoilage was very low or absent [96]. At the same time, the pH value increased for the control sample, indicating some degradation by growth and fermentation activity induced by microorganisms [97], like proteolysis of casein and lipolysis of fats [98,99].

The effect of the packaging films against *S. aureus*-inoculated cheese samples is presented in Table 4. As expected, the simple CMC0 has no antimicrobial activity against *S. aureus*, demonstrating a constant level of viable cells, around 5.5 log CFU/mL, with no significant growth over two weeks, but also with no reduction in the viable cells. Nevertheless, for all the samples packed in the composite films containing ZnO and TEO, the contamination level dropped below the detection limit for the *S. aureus* bacterial cells starting from the 3rd day. This behavior did not come as a surprise, given the strong antibacterial activity evidenced by the previous tests. However, the determination of the viable cells made on the first day, immediately after packing the samples, indicates a rapid decrease in the *S. aureus* counts, from 5.18 to 3.96 log CFU/mL in the case of the CTZ3 sample. The results suggest a stronger activity for the composite film with the highest TEO content, which can be explained by the rapid killing effect induced by the volatile components from the essential oil [100], while for the ZnO NPs, a longer contact time is required to generate the antibacterial action. Similar quick action was reported before for polymeric films with thymol [101], pepper essential oil [102] or plant extract [103]. Starch-based films with citric acid and sodium benzoate can decrease the *L. innocua* by 4.5 log CFU on cheese slices after 3 days [104]. On the other hand, for bilayer films, specially engineered for slow release, with low antimicrobial concentration (1000 ppm nisin), complete inhibition of *L. monocytogenes* was acquired only from day 9 [105].

Considering the properties of ZnO nanoparticles, the European Food Safety Authority (EFSA) concluded that it does not migrate in nanoform. At the same time, EFSA’s Scientific Committee on Food established a no observed adverse effect level for zinc at 50 mg/person per day, with a recommended upper limit of 25 mg/person per day. The safety assessment focuses on the migration of soluble ionic zinc [106]. However, within this study, we did not check the release of the ZnO NPs from the CMC matrix into the packed cheese.

## 4. Conclusions

Although numerous publications address CMC-based packaging films with ZnO NPs, relatively few studies have investigated the improvement of the antibacterial properties by adding a second natural antimicrobial agent, and none have addressed the specific combination of thyme essential oil and zinc oxide nanoparticles. The present study demonstrates that carboxymethyl cellulose can be successfully combined with zinc oxide nanoparticles and thyme essential oil to obtain biodegradable composite films with strong antibacterial activity and functional properties relevant for active food packaging. The antibacterial assays showed that the film composition can be adjusted in order to optimize the overall performance of the material while maintaining high antimicrobial efficacy. The obtained films rapidly reduced the viability of planktonic bacterial cells, with complete loss of viability observed after 3–4 h of contact for both *Escherichia coli* and *Staphylococcus aureus*. Preliminary tests on soft cheese further indicated the potential of these materials to extend refrigerated shelf life up to 14 days. *S. aureus* was not detected from the 3rd day in inoculated cheese slices packed in the composite films. These results support a complementary antibacterial action of ZnO and TEO within the composite matrix.

In addition to their biological activity, the composite films displayed improved mechanical and barrier performance by reducing water-vapor transfer and efficiently limiting the transmission of high-energy UV radiation associated with photo-oxidative degradation. The results demonstrate how antibacterial, mechanical and both barrier properties, UV and WVP, can be simultaneously enhanced through appropriate compositional design, allowing the tailoring of the final packaging film with the desired optimal properties. Overall, the developed CMC/ZnO/TEO films represent a promising biodegradable platform for active packaging applications intended for highly perishable food products.

## Figures and Tables

**Table 1:** Codification and composition for cellulose-based composite films.

Sample Code	Carboxymethyl Cellulose (CMC)	Thyme Essential Oil (TEO)	ZnO	Tween 80	Glycerol
CMC0	5 g	-	-		2 mL
CTZ1	5 g	0.5 mL	0.50 g	1 mL	2 mL
CTZ2	5 g	1 mL	0.50 g	1 mL	2 mL
CTZ3	5 g	2 mL	0.50 g	1 mL	2 mL
CTZ4	5 g	1 mL	0.25 g	1 mL	2 mL
CTZ5	5 g	1 mL	1 g	1 mL	2 mL

**Table 2:** Transmittance in UV and visible domains; barrier and mechanical properties of the films.

Sample Code	CMC0	CTZ1	CTZ2	CTZ3	CTZ4	CTZ5
UV-B	43.23	7.31	7.38	7.74	7.43	7.64
UV-A	76.21	6.10	6.15	6.56	6.15	6.27
Visible	97.30	90.78	90.16	89.27	90.59	88.80
Opacity	0.040 ± 0.000 ^a^	0.102 ± 0.002 ^b^	0.105 ± 0.004 ^b^	0.120 ± 0.006 ^c^	0.099 ± 0.002 ^b^	0.125 ± 0.008 ^c^
Thickness (mm)	0.30 ± 0.00 ^a^	0.41 ± 0.01 ^b^	0.43 ± 0.02 ^b^	0.41 ± 0.02 ^b^	0.43 ± 0.01 ^b^	0.41 ± 0.03 ^b^
WVP (10^−10^ g/Pa∙m∙s)	0.77 ± 0.02 ^a^	0.52 ± 0.02 ^b,c^	0.46 ± 0.01 ^d^	0.40 ± 0.02 ^e^	0.48 ± 0.01 ^b,d^	0.55 ± 0.03 ^c^
TS (MPa)	2.37 ± 0.10 ^a^	7.54 ± 0.26 ^b^	5.89 ± 0.42 ^c^	4.19 ± 0.17 ^d^	3.04 ± 0.45 ^a^	3.07 ± 0.51 ^a^
EB (%)	38.03 ± 4.24 ^a,b^	48.99 ± 1.41 ^c^	47.41 ± 0.71 ^c^	44.03 ± 4.04 ^a,c^	19.69 ± 2.83 ^d^	34.58 ± 4.95 ^b^
Young’s modulus (MPa)	3.94 ± 0.15 ^a^	11.32 ± 2.11 ^b^	9.28 ± 1.71 ^b,c^	8.36 ± 1.88 ^b,c^	7.22 ± 1.07 ^c^	37.11 ± 3.19 ^d^

Values are means ± standard deviation for *n* = 3 measurements. Different superscript letters in the same row indicate statistically significant differences between films (*p* < 0.05).

**Table 3:** The principal numerical data from thermal analysis.

Sample	T_1%_	T_5%_	T_10%_	Mass Loss RT-340 °C	Endo I	Endo II	Residual Mass (%)
CMC0	48 °C	102 °C	168 °C	75.79%	82.8 °C	256.8 °C	6.61%
CTZ1	89 °C	137 °C	181 °C	69.90%	110.8 °C	227.0 °C	12.10%
CTZ2	80 °C	132 °C	177 °C	68.65%	97.7 °C	246.0 °C	11.90%
CTZ3	69 °C	119 °C	167 °C	71.42%	92.0 °C	252.4 °C	10.99%
CTZ4	69 °C	130 °C	174 °C	73.06%	96.6 °C	276.7 °C	9.23%
CTZ5	78 °C	131 °C	178 °C	65.52%	100.3 °C	245.9 °C	15.30%

**Table 4:** Mass loss and *S. aureus* counts for soft cheese pieces coated with CMC0 and CTZ1-CTZ5 composite films.

Sample Code		CMC0	CTZ1	CTZ2	CTZ3	CTZ4	CTZ5
Mass loss (%) 14 days		30.57 ± 0.68 ^a^	21.60 ± 0.27 ^b^	19.33 ± 0.55 ^c^	16.49 ± 0.02 ^d^	20.07 ± 0.43 ^c^	19.87 ± 0.71 ^c^
*S. aureus* log CFU/mL	0 days	5.18 ± 0.11 ^a^	4.72 ± 0.12 ^b^	4.46 ± 0.15 ^b^	3.96 ± 0.10 ^c^	4.62 ± 0.08 ^b^	4.38 ± 0.18 ^b,c^
	3 days	5.23 ± 0.09 ^a^	N.D. ^b^	N.D. ^b^	N.D. ^b^	N.D. ^b^	N.D. ^b^
	7 days	5.61 ± 0.17 ^a^	N.D. ^b^	N.D. ^b^	N.D. ^b^	N.D. ^b^	N.D. ^b^
	14 days	5.77 ± 0.18 ^a^	N.D. ^b^	N.D. ^b^	N.D. ^b^	N.D. ^b^	N.D. ^b^

Values are means ± standard deviation for *n* = 3 measurements. Different superscript letters in the same row indicate statistically significant differences between films (*p* < 0.05).

## Data Availability

The original contributions presented in this study are included in the article. Further inquiries can be directed to the corresponding author.

## References

[1] Motelica L., Oprea O.-C. (2025). Antimicrobial Packaging: Materials and Applications. Reference Module in Food Science.

[2] Panahirad S., Dadpour M., Peighambardoust S.H., Soltanzadeh M., Gullon B., Alirezalu K., Lorenzo J.M. (2021). Applications of Carboxymethyl Cellulose- and Pectin-Based Active Edible Coatings in Preservation of Fruits and Vegetables: A Review. Trends Food Sci. Technol..

[3] Vuillet C., Guillard V., Angellier-Coussy H., Sudre G., Gouanvé F., Fleury E., Charlot A. (2026). Hydrophobization of Carboxymethyl Cellulose by Passerini Reaction: Towards Films with Improved Water Vapor Barrier Properties. J. Membr. Sci..

[4] Zou X.T., Zhao S.H., Xu K.W., Liu K., Yan C., Zhang X.J., Chen J., Cheng Y.L., Fang C.Q. (2025). Development and Characterization of Corn Starch-Based Films Enhanced with Chlorella Vulgaris Nanocellulose-Stabilized Pickering Emulsion of Zanthoxylum Bungeanum Essential Oil for Cherry Tomato Preservation. Int. J. Biol. Macromol..

[5] Yashwanth A., Huang R.D., Iepure M., Mu M.C., Zhou W.T., Kunadu A., Carignan C., Yegin Y., Cho D., Oh J.K. (2025). Food Packaging Solutions in the Post-per- and Polyfluoroalkyl Substances (PFAS) and Microplastics Era: A Review of Functions, Materials, and Bio-Based Alternatives. Compr. Rev. Food Sci. Food Saf..

[6] Wichaphian A., Yasan P., Pathom-aree W., Lumyong S., Suwannarach N., Kumla J., Chaipoot S., Hoijang S., Krasian T., Worajittiphon P. (2025). From Agricultural Waste to Active Films: Enhanced Crystallinity of Spent Mushroom Substrate-Derived Cellulose via Deep Eutectic Solvent-Based Microwave-Assisted Pretreatment and its Application in Reinforcing CMC-Based Composite Films. J. Agr. Food Res..

[7] Tohamy H.A.S. (2025). Novel Intelligent Naked-Eye Food Packaging pH-Sensitive and Fluorescent Sulfur, Nitrogen-Carbon Dots Biosensors for Tomato Spoilage Detection Including DFT and Molecular Docking Characterization. Int. J. Biol. Macromol..

[8] Suresh S.N., Senthilkumar P., Pushparaj C., Subramani R. (2025). Carboxy Methyl Cellulose/Carrageenan Composite Film Incorporated with Nanofibrils for Food Packaging Application. J. Appl. Polym. Sci..

[9] Gan H., Wang Y.L., Du J., Tao Y.H., Hu J.W., Lu J., Fu C.L., Xia X.D., Cao J.J., Wang H.S. (2026). Natural Pollen Stabilized Pickering Emulsions Incorporated in Carboxymethyl Cellulose-Based Active Packaging Films for Strawberry Preservation. Food Chem..

[10] Yousefi H., Fasihi M., Rasouli S. (2025). Tailoring Carboxymethyl Cellulose-Cased Food Packaging Films Blended with Polyvinyl Alcohol and Nano-MMT for Enhanced Performance and Shelf Life. Cellulose.

[11] Hashim S.B.H., Tahir H.E., Mahdi A.A., Shishir M.R.I., Marappan G., Abaker H.A.M., Khogly A.K.M., Aalim H., Karim N., Zhai X.D. (2026). A Hurdle Strategy to Develop Sugarcane Wax-Based O/W Emulsion for Enhancing the Functionality of Carboxymethyl Cellulose Sustainable Packaging Film. Int. J. Biol. Macromol..

[12] Oladzadabbasabadi N., Manamperi K., Dekiwadia C., Murdoch B.J., Hearn K., Ghasemlou M., Ivanova E.P., Adhikari B. (2026). Eco-Friendly Non-Isocyanate Polyurethane and Carboxymethyl Cellulose Composite Films Reinforced with Sodium Lignosulfonate for Sustainable Packaging Applications. Int. J. Biol. Macromol..

[13] Zielinska D., Wyrwas B., Lawniczak L., Borysiak S. (2025). Nanocellulose-Polypropylene Composites with Novel Antimicrobial Complex Salt. Polym. Compos..

[14] Vasile O.R., Serdaru I., Andronescu E., Trusca R., Surdu V.A., Oprea O., Ilie A., Vasile B.S. (2015). Influence of the Size and the Morphology of ZnO Nanoparticles on Cell Viability. Comptes Rendus Chim..

[15] Mobarraei M., Babaei S., Naseri M., Esmaeili M., Moosavi-Nasab M. (2025). Effect of Zinc Oxide Nanoparticles on the Physical, Mechanical, and Antibacterial Properties of Active Packaging Films Based on Gracilaria corticata Agar and Fish Skin Gelatin. Food Bioprocess. Technol..

[16] Lee S.W., Said N.S., Sarbon N.M. (2021). The Effects of Zinc Oxide Nanoparticles on the Physical, Mechanical and Antimicrobial Properties of Chicken Skin Gelatin/Tapioca Starch Composite Films in Food Packaging. J. Food Sci. Technol..

[17] Monsef A.S., Nemattalab M., Parvinroo S., Hesari Z. (2024). Antibacterial Effects of Thyme Oil Loaded Solid Lipid and Chitosan Nano-Carriers Against Salmonella Typhimurium and Escherichia Coli as Food Preservatives. PLoS ONE.

[18] Brezoiu A.M., Prundeanu M., Berger D., Deaconu M., Matei C., Oprea O., Vasile E., Negreanu-Pirjol T., Muntean D., Danciu C. (2020). Properties of *Salvia officinalis* L. and *Thymus serpyllum* L. Extracts Free and Embedded into Mesopores of Silica and Titania Nanomaterials. Nanomaterials.

[19] Satora P., Michalczyk M., Banas J. (2024). Impact of Thyme Essential Oil on the Aroma Profile and Shelf Life of Vacuum-Packed Minced Turkey Meat. Molecules.

[20] Aguado R.J., Saguer E., Fiol N., Tarrés Q., Delgado-Aguilar M. (2024). Pickering Emulsions of Thyme Oil in Water Using Oxidized Cellulose Nanofibers: Towards Bio-Based Active Packaging. Int. J. Biol. Macromol..

[21] Meeran M.F.N., Javed H., Al Taee H., Azimullah S., Ojha S.K. (2017). Pharmacological Properties and Molecular Mechanisms of Thymol: Prospects for Its Therapeutic Potential and Pharmaceutical Development. Front. Pharmacol..

[22] Kowalczyk A., Przychodna M., Sopata S., Bodalska A., Fecka I. (2020). Thymol and Thyme Essential Oil-New Insights into Selected Therapeutic Applications. Molecules.

[23] Gherasim O., Popescu R.C., Grumezescu V., Mogosanu G.D., Mogoanta L., Iordache F., Holban A.M., Vasile B.S., Birca A.C., Oprea O.C. (2021). MAPLE Coatings Embedded with Essential Oil-Conjugated Magnetite for Anti-Biofilm Applications. Materials.

[24] Mohamed S.A.A., Farouk A., Abdel-Razek A.G., Nashy E., El-Sakhawy M., Badr A.N. (2025). Carboxymethyl Cellulose/Shellac Composite Loaded with Pomegranate Extract and Jojoba Oil as Anti-Mycotic and Anti-Mycotoxigenic Food Packaging Materials. Sci. Rep..

[25] Motelica L., Vasile B.S., Ficai A., Surdu A.V., Ficai D., Oprea O.C., Andronescu E., Jinga D.C., Holban A.M. (2022). Influence of the Alcohols on the ZnO Synthesis and Its Properties: The Photocatalytic and Antimicrobial Activities. Pharmaceutics.

[26] Motelica L., Ficai D., Oprea O., Ficai A., Trusca R.D., Andronescu E., Holban A.M. (2021). Biodegradable Alginate Films with ZnO Nanoparticles and Citronella Essential Oil-A Novel Antimicrobial Structure. Pharmaceutics.

[27] Motelica L., Ficai D., Petrisor G., Oprea O.C., Trusca R.D., Ficai A., Andronescu E., Hudita A., Holban A.M. (2024). Antimicrobial Hydroxyethyl-Cellulose-Based Composite Films with Zinc Oxide and Mesoporous Silica Loaded with Cinnamon Essential Oil. Pharmaceutics.

[28] (2019). Plastics—Determination of Tensile Properties—Part 1: General Principles.

[29] (2018). Plastics—Determination of Tensile Properties—Part 3: Test Conditions for Films and Sheets.

[30] Zhang J.H., Su J.C., Liu X.Y., Chen M., Bai B.Q., Yang Y.K., Fan S.H., Bo T. (2025). Preparation and Characterization of Carboxymethyl Cellulose-Based Edible Thin Films Loaded with Rosmarinic Acid and Citral Nanoparticles. Carbohydr. Polym. Technol..

[31] Thiyagamoorthy U.M., Sadayandi G., Jeyaraj S., Thangarasu S., Wadaan M.A., Baabbad A., Vafaeva K.M., Packialakshmi J.S. (2024). Exploring the Efficacy of Various Essential Oils in Chitosan-Based Composite Biopolymer Films for Food Packaging. Polym. Adv. Technol..

[32] Zikeli F., Vinciguerra V., Sennato S., Mugnozza G.S., Romagnoli M. (2020). Preparation of Lignin Nanoparticles with Entrapped Essential Oil as a Bio-Based Biocide Delivery System. Acs Omega.

[33] Mansur A.A.P., de Carvalho F.G., Mansur R.L., Carvalho S.M., De Oliveira L.C., Mansur H.S. (2017). Carboxymethylcellulose/ZnCdS Fluorescent Quantum Dot Nanoconjugates for Cancer Cell Bioimaging. Int. J. Biol. Macromol..

[34] Joyline G., Gachoki K.P., Ngure G.A., Nyambura N.C., Shigwenya M.E. (2023). High Swelling Carboxymethyl Cellulose Synthesized from Coconut Fibers. J. Nat. Fibers.

[35] Shlosman K., Rein D.M., Shemesh R., Cohen Y. (2024). Lyophilized Emulsions of Thymol and Eugenol Essential Oils Encapsulated in Cellulose. Polymers.

[36] Shlosman K., Rein D.M., Shemesh R., Koifman N., Caspi A., Cohen Y. (2023). Encapsulation of Thymol and Eugenol Essential Oils Using Unmodified Cellulose: Preparation and Characterization. Polymers.

[37] Chen L., Zhou H.J., Hao L., Chen H.Y., Zhou X.H. (2019). Soy Protein Isolate-Carboxymethyl Cellulose Conjugates with pH Sensitivity for Sustained Avermectin Release. R. Soc. Open Sci..

[38] Dogaru A.I., Oprea O.C., Isopencu G.O., Banciu A., Jinga S.I., Busuioc C. (2025). Bacterial Cellulose-Based Nanocomposites for Wound Healing Applications. Polymers.

[39] de la O-cuevas E., Gallegos-Flores P., Ortega-Sigala J.J., Tototzintle-Huitle H., Saniger J.M., Esparza-Ibarra E.L. (2025). Biophysical and Structural Modifications on Human Erythrocytes Induced by Ethanolic Extracts of *Ruta graveolens*, *Artemisia ludoviciana*, and *Lippia graveolens*: A Study by ATR-FTIR Spectroscopy. RSC Adv..

[40] Catauro M., Bollino F., Tranquillo E., Sapio L., Illiano M., Caiafa I., Naviglio S. (2017). Chemical Analysis and Anti-Proliferative Activity of Campania Thymus Vulgaris Essential Oil. J. Essent. Oil Res..

[41] Markovic M.M., Anicic B., Lazarevic M., Karisik M.J., Mitic D., Milovanovic B., Ivanovic S., Pecinar I., Petrovic M., Petrovic M. (2025). Cytotoxic Effects of *Thymus serpyllum* L. and *Mentha* × *piperita* L. Essential Oils on Basal Cell Carcinoma—An In Vitro Study. Life.

[42] Vedovello P., Paiva R.S., Bortoletto-Santos R., Ribeiro C., Putti F.F. (2025). The Effect of the Conformation Process on the Physicochemical Properties of Carboxymethylcellulose-Starch Hydrogels. Gels.

[43] Busuioc C., Isopencu G., Banciu A., Banciu D.D., Oprea O., Mocanu A., Deleanu I., Zaulet M., Popescu L., Tanasuica R. (2022). Bacterial Cellulose Hybrid Composites with Calcium Phosphate for Bone Tissue Regeneration. Int. J. Mol. Sci..

[44] Sasaki J.C.D., Su Y.J., Spinosa W.A., Lopes P.E.D., Burd B.S., Scontri M., Tanaka J.L., Gonçalves R.P., Bridi F.B., dos Santos L.S. (2025). Eco-Sustainable, Edible, Biodegradable and Antioxidant Pectin and Bacterial Cellulose Films Loaded with Coconut Oil for Strawberry Preservation. Int. J. Biol. Macromol..

[45] Matwijczuk A., Oniszczuk T., Matwijczuk A., Chrusciel E., Kocira A., Niemczynowicz A., Wójtowicz A., Combrzynski M., Wiacek D. (2019). Use of FTIR Spectroscopy and Chemometrics with Respect to Storage Conditions of Moldavian Dragonhead Oil. Sustainability.

[46] Zheng S.W., Rupa E.J., Chokkalingam M., Piao X.M., Han Y.X., Ahn J.C., Nahar J., Kong B.M., Kwak G.Y., Kim J.H. (2022). Photocatalytic Activity of Orchid-Flower-Shaped ZnO Nanoparticles, toward Cationic and Anionic Dye Degradation under Visible Light, and Its Anti-Cancer Potential. Coatings.

[47] Romulo A., Anjani V.S., Wardana A.A. (2024). Enhancing Antimicrobial Activity of Thyme Essential Oil Through Cellulose Nano Crystals-Stabilized Pickering Emulsions. Foods.

[48] Zhou R., Guo C.B., Li Q., Li Z.L., Fan W.D., Chen X., Dai J., Zhang Q. (2026). Development of Bacterial Cellulose-Based Films Incorporated with Epigallocatechin-3-Gallate for Active Food Packaging. Foods.

[49] Motelica L., Ficai D., Oprea O.C., Ficai A., Ene V.L., Vasile B.S., Andronescu E., Holban A.M. (2021). Antibacterial Biodegradable Films Based on Alginate with Silver Nanoparticles and Lemongrass Essential Oil-Innovative Packaging for Cheese. Nanomaterials.

[50] Zafar A., Khosa M.K., Noor A., Qayyum S., Saif M.J. (2022). Carboxymethyl Cellulose/Gelatin Hydrogel Films Loaded with Zinc Oxide Nanoparticles for Sustainable Food Packaging Applications. Polymers.

[51] Abu Rayhan M., Hossain M.M., Rahman T., Mia M.S., Zzaman W. (2026). Development of UV-Resistant Carboxymethyl Cellulose/ZnO-NPs Based Nanocomposite Films Derived from Zea Mays Husk and Reinforced with Ananas Comosus Peel Extract for Active Food Packaging. Carbohydr. Polym. Technol..

[52] Fierascu I., Fierascu R.C., Fistos T., Motelica L., Oprea O., Nicoara A., Ficai A., Stirban A., Zgarciu M.S. (2021). Non-Invasive Microanalysis of a Written Page from the Romanian Heritage “The Homiliary of Varlaam (*Cazania lui Varlaam*)”. Microchem. J..

[53] Motelica L., Popescu A., Razvan A.G., Oprea O., Trusca R.D., Vasile B.S., Dumitru F., Holban A.M. (2020). Facile Use of ZnO Nanopowders to Protect Old Manual Paper Documents. Materials.

[54] Gheorghita D., Antoniac I., Moldovan H., Antoniac A., Grosu E., Motelica L., Ficai A., Oprea O., Vasile E., Ditu L.M. (2023). Influence of Lavender Essential Oil on the Physical and Antibacterial Properties of Chitosan Sponge for Hemostatic Applications. Int. J. Mol. Sci..

[55] Ciocirlan O., Gavrila A., Isopencu G., Motelica L., Oprea O.C., Nicoara A.I., Sima S., Stanescu P. (2025). Formulation and Characterization of Chitosan Films Incorporating Hawthorn Polyphenolic Extracts via Natural Deep Eutectic Solvents. Polymers.

[56] Motelica L., Ficai D., Ficai A., Trusca R.D., Ilie C.I., Oprea O.C., Andronescu E. (2020). Innovative Antimicrobial Chitosan/ZnO/Ag NPs/Citronella Essential Oil Nanocomposite—Potential Coating for Grapes. Foods.

[57] Dumitru C.D., Ilie C.I., Neacsu I.A., Motelica L., Oprea O.C., Ripszky A., Pituru S.M., Balasea B.V., Marinescu F., Andronescu E. (2025). Antimicrobial Composite Films Based on Alginate-Chitosan with Honey, Propolis, Royal Jelly and Green-Synthesized Silver Nanoparticles. Int. J. Mol. Sci..

[58] Pei R., Lu H., Wang F., Ma R.R., Tian Y.Q. (2023). The Fluorescence Response of Four Crystalline Starches According to Ultrasound-Assisted Starch-Salicylic Acid Inclusions. Foods.

[59] Gu Y.A., Zhao Z., Su H.F., Zhang P.F., Liu J.K., Niu G.L., Li S.W., Wang Z.Y., Kwok R.T.K., Ni X.L. (2018). Exploration of Biocompatible AIEgens from Natural Resources. Chem. Sci..

[60] Luo J.D., Xie Z.L., Lam J.W.Y., Cheng L., Chen H.Y., Qiu C.F., Kwok H.S., Zhan X.W., Liu Y.Q., Zhu D.B. (2001). Aggregation-Induced Emission of 1-Methyl-1,2,3,4,5-Pentaphenylsilole. Chem. Commun..

[61] Cai X.M., Lin Y.T., Li Y., Chen X.F., Wang Z.Y., Zhao X.Q., Huang S.L., Zhao Z., Tang B.Z. (2021). BioAIEgens Derived from Rosin: How Does Molecular Motion Affect their Photophysical Processes in Solid State?. Nat. Commun..

[62] Zhang H.K., Tang B. (2021). Through-Space Interactions in Clusteroluminescence. JACS Au.

[63] Yuan W.Z., Zhang Y.M. (2017). Nonconventional Macromolecular Luminogens with Aggregation-Induced Emission Characteristics. J. Polym. Sci. Pol. Chem..

[64] Castellan A., Ruggiero R., Frollini E., Ramos L.A., Chirat C. (2007). Studies on Fluorescence of Cellulosics. Holzforschung.

[65] Gong Y.Y., Tan Y.Q., Mei J., Zhang Y.R., Yuan W.Z., Zhang Y.M., Sun J.Z., Tang B.Z. (2013). Room Temperature Phosphorescence from Natural Products: Crystallization Matters. Sci. China Chem..

[66] Voicu G., Oprea O., Vasile B.S., Andronescu E. (2013). Photoluminescence and Photocatalytic Activity of Mn-Doped Zno Nanoparticles. Dig. J. Nanomater. Bios.

[67] Zorca A.G., Chira N., Isopencu G.O., Busuioc C., Oprea O.C., Covaliu-Mierla C.I., Cîrîc A., Toader G., Deleanu I.M. (2025). Fabrication of Functional Edible Packaging Materials Based on Carboxymethyl Cellulose and Pomelo Peel Powder Supplemented with Nisin as Active Compound. Carbohydr. Polym. Technol..

[68] Mohammadi H., Kamkar A., Misaghi A. (2018). Nanocomposite Films Based on CMC, Okra Mucilage and ZnO Nanoparticles: Physico Mechanical and Antibacterial Properties. Carbohydr. Polym..

[69] Isopencu G., Deleanu I., Busuioc C., Oprea O., Surdu V.A., Bacalum M., Stoica R., Stoica-Guzun A. (2023). Bacterial Cellulose-Carboxymethylcellulose Composite Loaded with Turmeric Extract for Antimicrobial Wound Dressing Applications. Int. J. Mol. Sci..

[70] Balen R., da Costa W.V., Andrade J.D., Piai J.F., Muniz E.C., Companhoni M.V., Nakamura T.U., Lima S.M., Andrade L.H.D., Bittencourt P.R.S. (2016). Structural, Thermal, Optical Properties and Cytotoxicity of PMMA/ZnO Fibers and Films: Potential Application in Tissue Engineering. Appl. Surf. Sci..

[71] Nassif R.A., Hilal R.H., Salih R.M. (2024). Preparation and Characterisation of Polymer Blends Reinforced with Nano-ZnO and Study the Thermal and Electrical Properties for Industrial Applications. Kuwait J. Sci..

[72] Sheikh M., Asghari M., Afsari M. (2018). Effect of Tiny Amount of Zinc Oxide on Morphological and Thermal Properties of Nanocomposite PEBA Thin Films. Alex. Eng. J..

[73] Farahani F.K., Oromiehi A.R., Sharifan A., Farahani Z.K. (2025). Edible Films Solution Developed from Carboxymethyl Cellulose/Sesame Oil: Physical, Mechanical and Microbial Attributes of Films. Appl. Food Res..

[74] Salama H.E., Aziz M.S.A. (2024). Optimized UV-Barrier Carboxymethyl Cellulose-Based Edible Coatings Reinforced with Green Synthesized ZnO-NPs for Food Packaging Applications. Polym. Bull..

[75] Tavares K.M., de Campos A., Luchesi B.R., Resende A.A., de Oliveira J.E., Marconcini J.M. (2020). Effect of Carboxymethyl Cellulose Concentration on Mechanical and Water Vapor Barrier Properties of Corn Starch Films. Carbohydr. Polym..

[76] Spinei M., Oroian M., Ursachi V.F. (2024). Characterization of Biodegradable Films Based on Carboxymethyl Cellulose and Citrus Pectin Films Enriched with Bee Bread Oil and Thyme Oil. LWT-Food Sci. Technol..

[77] Doveri L., Fernandez Y.A.D., Dacarro G., Grisoli P., Milanese C., Urena M., Sok N., Karbowiak T., Pallavicini P. (2025). Active Pectin/Carboxymethylcellulose Composite Films for Bread Packaging. Molecules.

[78] Vyas A., Ng S.P., Fu T., Anum I. (2025). ZnO-Embedded Carboxymethyl Cellulose Bioplastic Film Synthesized from Sugarcane Bagasse for Packaging Applications. Polymers.

[79] Yousefi N., Zahedi Y., Yousefi A., Hosseinzadeh G., Jekle M. (2024). Development of Carboxymethyl Cellulose-Based Nanocomposite Incorporated with ZnO Nanoparticles Synthesized by Cress Seed Mucilage as Green Surfactant. Int. J. Biol. Macromol..

[80] Anwar M.M., Aly S.S.H., Nasr E.H., El-Sayed E.R. (2022). Improving Carboxymethyl Cellulose Edible Coating Using ZnO Nanoparticles from Irradiated Alternaria Tenuissima. Amb. Express.

[81] Mei L.Y., Shi L.X., Song X.L., Liu S., Cheng Q., Zhu K., Zhuge R.X. (2021). Characterization of Carboxymethyl Cellulose Films Incorporated with Chinese Fir Essential Oil and Their Application to Quality Improvement of Shine Muscat Grape. Coatings.

[82] Wang H., Han P., Zhao Y.H., Lu L.J., Qi W.H., Zhao K.X., Shu Y., Zhang Z.S. (2025). Preparation and Characteristics of Carboxymethyl Cellulose-Based Films Embedding Cinnamon Essential Oil and Their Application on Mutton Preservation. Front. Nutr..

[83] How Y.H., Lim E.M.Y., Kong I.N., Kee P.E., Pui L.P. (2024). Development of Carboxymethyl Cellulose-Chitosan Based Antibacterial Films Incorporating a Persicaria Minor Huds. Essential Oil Nanoemulsion. Sustain. Food Technol..

[84] Nahas E.O., Andrade G.S.S., Lopes M.S., Silva E.K. (2025). Eco-Friendly Carboxymethyl Cellulose Films Incorporated with Phenolic Compounds from Hydrodistillation Wastewater of Rosemary Essential Oil. Int. J. Biol. Macromol..

[85] Guo X.G., Chen B.R., Wu X.L., Li J.M., Sun Q. (2020). Utilization of Cinnamaldehyde and Zinc Oxide Nanoparticles in a Carboxymethylcellulose-Based Composite Coating to Improve the Postharvest Quality of Cherry Tomatoes. Int. J. Biol. Macromol..

[86] Jin S.E., Jin H.E. (2021). Multiscale Metal Oxide Particles to Enhance Photocatalytic Antimicrobial Activity against Escherichia coli and M13 Bacteriophage under Dual Ultraviolet Irradiation. Pharmaceutics.

[87] Abebe B., Zereffa E.A., Tadesse A., Murthy H.C.A. (2020). A Review on Enhancing the Antibacterial Activity of ZnO: Mechanisms and Microscopic Investigation. Nanoscale Res. Lett..

[88] Zhu X.Y., Wang J., Cai L., Wu Y., Ji M.H., Jiang H.J., Chen J. (2022). Dissection of the Antibacterial Mechanism of Zinc Oxide Nanoparticles with Manipulable Nanoscale Morphologies. J. Hazard. Mater..

[89] Valliammai A., Selvaraj A., Yuvashree U., Aravindraja C., Pandian S.K. (2020). sarA-Dependent Antibiofilm Activity of Thymol Enhances the Antibacterial Efficacy of Rifampicin Against Staphylococcus aureus. Front. Microbiol..

[90] Tokam C.R.K., Ndezo B.B., Boulens N., Allémann E., Delie F., Dzoyem J.P. (2023). Antibiofilm Activity and Synergistic Effects of Thymol-Loaded Poly (Lactic-Co-Glycolic Acid) Nanoparticles with Amikacin against Four Salmonella enterica Serovars. Can. J. Infect. Dis. Med..

[91] Zhu Z., Min T.T., Zhang X.J., Wen Y.Q. (2019). Microencapsulation of Thymol in Poly(lactide-co-glycolide) (PLGA): Physical and Antibacterial Properties. Materials.

[92] Qureshi K.A., Parvez A. (2026). Antimicrobial and Antibiofilm Evaluation of Thymol, Sodium Azide, and Sodium Lauryl Sulfate Against Multidrug-Resistant Pathogens: An Integrated Experimental and Computational Study. PLoS ONE.

[93] Motelica L., Vasile B.-S., Ficai A., Surdu V.-A., Ficai D., Oprea O.C., Andronescu E., Mustățea G., Ungureanu E.L., Dobre A.A. (2023). Antibacterial Activity of Zinc Oxide Nanoparticles Loaded with Essential Oils. Pharmaceutics.

[94] Youssef A.M., Assem F.M., El-Sayed H.S., El-Sayed S.M., Elaaser M., Abd El-Salam M.H. (2020). Synthesis and Evaluation of Eco-Friendly Carboxymethyl Cellulose/Polyvinyl Alcohol/CuO Bionanocomposites and Their Use in Coating Processed Cheese. RSC Adv..

[95] Yildirim-Yalcin M., Tornuk F., Toker O.S. (2022). Recent Advances in the Improvement of Carboxymethyl Cellulose-Based Edible Films. Trends Food Sci. Technol..

[96] Azhdari S., Moradi M. (2022). Application of Antimicrobial Coating Based on Carboxymethyl Cellulose and Natamycin in Active Packaging of Cheese. Int. J. Biol. Macromol..

[97] Nottagh S., Hesari J., Peighambardoust S.H., Rezaei-Mokarram R., Jafarizadeh-Malmiri H. (2020). Effectiveness of Edible Coating Based on Chitosan and Natamycin on Biological, Physico-Chemical and Organoleptic Attributes of Iranian Ultra-Filtrated Cheese. Biologia.

[98] De Wit M., Osthoff G., Viljoen B.C., Hugo A. (2005). A Comparative Study of Lipolysis and Proteolysis in Cheddar Cheese and Yeast-Inoculated Cheddar Cheeses During Ripening. Enzym. Microb. Technol..

[99] Collins Y.F., McSweeney P.L.H., Wilkinson M.G. (2003). Lipolysis and Free Fatty Acid Catabolism in Cheese: A Review of Current Knowledge. Int. Dairy. J..

[100] Doukaki A., Frantzi A., Xenou S., Schoina F., Katsimperi G., Nychas G.J., Chorianopoulos N. (2026). Antimicrobial Effect of Oregano Essential Oil in Na-Alginate Edible Films for Shelf-Life Extension and Safety of Feta Cheese. Pathogens.

[101] Chang S., Nafchi A.M., Baghaie H. (2021). Development of an Active Packaging Based on Polyethylene Containing Linalool or Thymol for Mozzarella Cheese. Food Sci. Nutr..

[102] Dannenberg G.D., Funck G.D., Cruxen C.E.D., Marques J.D., da Silva W.P., Fiorentini A.M. (2017). Essential Oil from Pink Pepper as an Antimicrobial Component in Cellulose Acetate Film: Potential for Application as Active Packaging for Sliced Cheese. LWT-Food Sci. Technol..

[103] Mahmud J., Heredia J., Sharaby M.R., Jaiswal L., Salmieri S., Moosavi S.E., Lacroix M. (2026). Development of Bioactive Carboxymethyl Cellulose-Based Films via Dual Crosslinking with Citric Acid and X-Ray Irradiation. Foods.

[104] de Moraes J.O., Hilton S.T., Moraru C. (2020). The Effect of Pulsed Light and Starch Films with Antimicrobials on *Listeria innozcua* and the Quality of Sliced Cheddar Cheese During Refrigerated Storage. Food Control.

[105] Divsalar E., Tajik H., Moradi M., Forough M., Lotfi M., Kuswandi B. (2018). Characterization of Cellulosic Paper Coated with Chitosan-Zinc Oxide Nanocomposite Containing Nisin and Its Application in Packaging of UF Cheese. Int. J. Biol. Macromol..

[106] Bolognesi C., Castle L., Cravedi J.-P., Engel K.-H., Franz R., Fowler P., Grob K., Gürtler R., Husøy T., Kärenlampi S. (2016). Safety Assessment of the Substance Zinc Oxide, Nanoparticles, for Use in Food Contact Materials. EFSA J..

